# Finishing, Fillings, Etc.

**Published:** 1859-02

**Authors:** A. F. Ennis

**Affiliations:** Plymouth, N. C.


### FINISHING, FILLINGS, ETC.

BY A. F. ENNIS, PLYMOUTH, N. C.

Editor of Reporter—*Dear Sir*:—I send you a few ideas important to every member of our profession to observe, if you find them worthy of insertion, they are at your disposal.

The necessity of great care in completing the process of filling after consolidating a plug, all will admit.

There are many good operators who are neglectful in this most important and (as I conceive) indispensable duty, of thoroughly burnishing fillings, until they present a high polish and luster sufficient to reflect your image. The most successful operators in dental surgery in America, among whom I will mention, Professor C. A. Harris, of the Baltimore College of Dental Surgery; the lamented Dr. John Harris, deceased; Dr. Badger, of Nashville, Tenn., and a host of others were particular in regard to this item, and, indeed, I esteem the proper finish a sine qua non, upon which the future success depends. What would you think of a cabinet maker who would offer to sell you a roughly dressed piece of mahogany furniture. The furniture may be well put together, but lacking the finish—it tells unfavorably upon the workman. Likewise, a filling may be well put into the cavity and properly consolidated, but without the finishing stroke, characteristic of the accomplished artizan, the eye is not pleased. I have often observed a negligence among some operators in this particular, and, therefore, I wish to jog the memory of members of the profession to the importance of doing what they do, in the most thorough manner. An half hour can not be better spent than to condense, consolidate and burnish, until a high finish is obtained on a filling—it will amply repay the operator, and speak volumes toward the adornment of his path in life. The same care is essential in completing other operations; for instance, after separating, use a worn-out file or emery paper and polish. These, though small items, are indispensable steps to success. Particles of food are prevented from lodging on the surface of the enamel or dentine, and the tooth is secured against further decay, when well filled, provided the patient observes proper care in keeping the surface clean, hence, dentists should direct their patients to use floss-silk to cleanse the teeth.

The dentist can not be too particular after removing salivary calculus, to apply crocus, pummice-stone or other polishing powder, to leave a high polish. This course removes all traces of scratches, roughness, or particles of tartar that adhere to the enamel, and causes a highly polished, natural glazed surface which does not favor the accumulation of calcarious deposits. If the patient will follow the advice of his dentist, in observing the rules of dental hygiene and therapeutics, he will not fail to enjoy the luxury of a good Dental Organism.

Acids should, under no circumstances, be used about the natural organs, in our practice.

---

The following lecture was delivered Dec. 23, in the Ohio Dental College, Cincinnati, by Albany Packham, D. M. C. (at the hour which should have been occupied by one of the Faculty, but who failed to "come to time,") wherein he faithfully portrayed the several peculiarities of the profession: for instance, the round, soft and *repeating* style of Chapman; the excited, quick, railroad speed of Taft; the clear, smooth, plain, yet forcible manner of Watt; the grand and sure originality of Richardson; the loud tone, and gesticulating action of Smith; the dignified and stately grandeur of Taylor.

GENTLEMEN:—You have requested me to deliver before you a lecture upon Mrs. Partington, but having studied the *classical* languages in preference to my own, I am afaid you will not understand *my* English, so as to appreciate my lecture, if I deliver it in that language; still, if you will overlook my imperfections of speech, I will proceed. (Go on! Go on!)

Gentlemen, go-a-head it is, then—I am very glad to see so many representatives of the Partington family present—I will, therefore, make a few brief remarks about myself before I proceed with the Old Lady. In the first place, I am like unto a rolling stone which has gathered no moss, for I have been rolling from place to place, till I have nary-a-moss sticking to me. Let me then, impress upon your minds, the absolute necessity of locating yourself in some likely spot, where human pearls are scarce, and stay there if you want to gather the moss. I started in life with as fair a prospect of success as any one of you now present, but I wandered from place to place, until I have traveled upwards of six thousand miles, and spent upwards of three thousand dollars, then I could not roll my stones any farther. Gentlemen, when you are once settled and doing well, yet think you can do better by *shift*ing your shingle, don't do it! but think of me! and my word for it, you will stay--that is, if your neighbors will let you. (Cheers.)

Gentlemen, This, I believe, is the only instance when an honor like the present, has been conferred on a janitor, lecturing (by the request of, and before the class) since the time when Aristotle, Esculapius, and the ancient faculty used to lecture before the students in the shady groves of Athens, Rome and Greece, for the janitor's duty at that time was to sweep up the fallen leaves, for the students to sit upon, and also to keep the nurse girls away. This, I say, is an honor never before conferred upon a janitor, and as such I thank you, not in the common slang of the day, (from the bottom of my heart,) for I believe my heart knows nothing about it, but I thank you, gentlemen, from the center of my brain. (Cheers.)

There is something about me that pleases you, else why this kind notice, which each of you have always shown me, others also, have been struck by my classical appearance, for during last summer, while my friend, Dr. Ruth, there on my right, had charge of the Infirmary, a lady came *in* to have a tooth *out*—she was placed in the operating chair—she was very nervous—for seven times did he take the forceps and approach her capacious aperture, containing the masticating machinery, and seven times did she close it with a sound like that of a snapping-turtle, suddenly she caught a glimpse of your humble servant, her eyes brightened up instantly, at my prepossessing appearance, and pointing at me, said if the *professor* there will hold me *in* I will have it *out*. I did as the lady requested, and the doctor extracted a three legged stool, which I suppose a cow had previously kicked there, when she was milking. At another time, that very nice, ancient but ignorant old lady, Mrs. Partington, came to the College, with her young hopeful son Ike, and wanted to know if I was not the professor, over all the Faculties. I did not deny the soft impeachment, but nodded my head in the affirmative—she said, that seeing our printed circulars scattered all over creation, wherein it stated that all those applying for work at the Infirmary, would be treated with all the kindness and respect that they would receive in a private office. I said certainly, madam, it's so! She then wanted me to *give* Ike a job of work, and five dollars per week. I explained to her the mistake she was under, by stating we expected patients to give us a job of dentistry-work. She then wished to know if Ike was disqualified to learn dysentery. I told her I could soon tell her, by examining him in arithmetic and geography. She told Ike to stand up, put his hands behind him, and answer me like a man! I asked him what addition meant? Ike said applying the battery in pulling a *fang*, in addition to the screw forceps, (good!) What does multiplication mean? He promptly replied, multiplying the pain in toothache, by the application of Dr. Branch's Patent Salt and Ice Bag, (good again.) What is division? He replied without hesitation, dividing the jaw, while extracting a tooth, (good again.) I asked him only one question in geography, and that was how far he expected his practice to extend? He said he would not be content unless his practice extended from the frozen ice-burghs of the North Pole to the warm-hearted sunny south, and from the eastern land of nasal-twangs to the other side of the setting sum. I turned to the anxious mother and said, Ike's examination proved him disqualified to study dysentery, and as she was very anxious to see him try his hand. I told him to commence operations, (she whispered in my ear that Ike had one operation that morning,) by extracting a canine tooth, he looked in his Latin for the word *canine*, then darted into the street after a dog, returned and placed it in the operating chair, seized a forcep, asked the poor brute if he would have the battery? He barked out, no! as plain as bark could. He then asked it to have Dr. Branch's Patent Ice and Salt Bag? (Applause.) A bark and a growl, so savage was the answer, that Ike dropped the forceps in alarm, and the brute escaped. I informed him it was not dog teeth I meant when I said canine. He looked at me with his nose turned up, in derision of my ignorance, and muttered something about my knowing nothing about Latin. I handed him a skull and told him to extract the cus-pidatis. Poor Mrs. Partington, with tears in her eyes, exclaimed in a rage, (her voice hissing like a hot casting, thrown into the water,) Ike shan't *detract*, nor *cuss-his-daddy*, for he was a good daddy to him, and died when he was only two months old—I mean Ike was two months old, and not his daddy—and before I could explain, she put up her spectacles, and dragged her young hopeful out of the College, in disgust with dentists in general, and dysentery in particular. (Cheers.)

Gentlemen, one of the junior members of the class, told me in confidence and friendship, that Mrs. Partington was really dead, and not to make myself ridiculous by saying she came into the Infirmary. Gentlemen, there are a few more left, and a great many son Ikes, and that young man just mentioned is one, and unless he greatly improves, he will do worse than his name-sake did with his dog patient. Why, every town in the world has a Mrs. Partington in their midst, any one of you (if you except that young man, for he did not know his own mother) can always distinguish the old lady from the rest of the human family, by these few peculiarities—she invariably confounds verbs, moods and tenses, calling things by the wrong names, and giving the most absurd pronunciations to words—she is a kind, good, motherly old soul, her hand in her pocket at the first sigh of distress, either real or imaginary, it's all the same to her, (the reason there are so many professional beggars, is because there are so many Mrs. Partingtons,) she always wears spectacles, and when excited, loses them sure, she then commences searching for them in her bureau drawers, inside of her Bible, in her work basket, in her pocket, and even in her button bag, at last finds them above her nose, but *knows* nothing about them, until told of the circumstance. Mrs. P. never was guilty of giving her son a clean shirt to put *on*, having a button *off*, neither does she give him his stockings with a hole in them, except the one he *put his foot into*, (nothing personal, gentlemen, although we all know some of you had a *hand in it* the other day,) she is also very firm and headstrong in her belief of whatever she has set her mind upon, for she still insists that her son Ike was subject to the *inflatation* of the *growels* every time he went to see the *inflammation* of the balloon, and when he was undergoing the painful operation of cutting his *cotemporary* teeth, he had fits and *revolutions*. Such then is Mrs. Partington, and with all her faults, I love her still. (Applause.)

Gentlemen, from this time forth, I shall endeavor so to shape my future conduct, that each and every one of you, when far away, will be proud to acknowledge me among your correspondents, and own up to your friends, that Albany Packham was once your Demonstrator of MANUAL COMBUSTION, IN THE OHIO DENTAL COLLEGE. Again, gentlemen, I thank you from the center of my brain, for your kind attention and stunning applause.

